# Working status of caregivers for people with dementia: Analysis data from a Japanese Nationwide Survey

**DOI:** 10.1371/journal.pone.0232787

**Published:** 2020-05-29

**Authors:** Norio Sugawara, Norio Yasui-Furukori, Kazushi Maruo, Kazutaka Shimoda, Tomiki Sumiyoshi

**Affiliations:** 1 Department of Clinical Epidemiology, Translational Medical Center, National Center of Neurology and Psychiatry, Kodaira, Tokyo, Japan; 2 Department of Psychiatry, Dokkyo Medical University School of Medicine, Mibu, Tochigi, Japan; 3 Department of Neuropsychiatry, Hirosaki University School of Medicine, Hirosaki, Aomori, Japan; 4 Faculty of Medicine, University of Tsukuba, Tsukuba, Ibaraki, Japan; University of Technology Sydney, AUSTRALIA

## Abstract

**Background:**

The prevalence of dementia has increased rapidly in Japan, while the proportion of the population accounted for by working-age individuals is facing a sharp decline. Optimizing the balance between work and caregiving for persons with dementia is a major public health issue.

**Aims:**

Using a nationally representative sample, this study investigated the working status of caregivers (CGs) for elderly people (care recipients) with dementia (CRDs) and assessed the effects of sociodemographic factors on this status.

**Methods:**

Data were obtained from the 2013 Comprehensive Survey of the Living Conditions for CRDs and CGs (the latter aged 65 years or less). Individual data of CRDs and CGs were linked, and 452 pairs were extracted. The Japanese version of the Kessler 6 (K6) with a cut-off point of 13 was used to assess general psychological distress among CGs. Multivariable logistic regression analysis with the forward selection method was used to identify the predictors of their working status.

**Results:**

Overall, the mean age of CGs was 57.1 ± 6.8 years, with 57.5% (260/452) performing paid work. Male sex, higher educational attainment, and having their own house were associated with having paid work for CGs, while higher age, spending almost all day performing nursing care, and participation in helping with toilet activities and laundry were associated with not performing paid work.

**Conclusions:**

Several sociodemographic factors, including nursing care-related factors, are associated with the employment status of CGs. Further research should examine detailed information on CRDs’ activities of daily living, behavioral and psychological symptoms, medical service use, and social support to strengthen the system of supportive services for both CRDs and their CGs.

## Introduction

Dementia is a progressive disorder that causes a decline in several cognitive domains (often memory and at least 1 other domain, such as executive function, language, attention, visuospatial abilities, or other domains) and is severe enough to affect daily functioning and independence [1]. The prevalence of dementia in Japan has increased at a rapid pace because of the aging population and the increased average life span [2]. Most elderly Japanese hope to receive home nursing care rather than institutional or medical care [3]. Although family caregivers (CGs) play an essential role in supporting the well-being and care of older people, caregiving for elderly people with dementia places a considerable burden on family CGs. This burden is not restricted to only the economic perspective [4, 5], such as the loss of productivity, but also affects psychological distress, including anxiety or depression [6, 7]. Furthermore, family CGs often encounter unmet needs with regard to formal care and feel abandoned and unrecognized by the health care system [8, 9]. Traditionally, nonworking family members have taken the role of family CGs. However, the recent trend shows an increasing number of primary CGs who maintain paid employment due to the fact that there are fewer family members per household. Because the proportion of the population who are working-age in Japan is facing a steep decline [10], optimizing the balance between work and caregiving for persons with dementia is a public health issue.

To date, several studies have investigated the association between CGs' mental health and caregiving for older relatives among working individuals in Japan [5, 11–13]. However, few studies have clarified the factors associated with working status among working-age family CGs for people with dementia. In addition, family CGs' roles vary based on by the care needs of elderly people and often includes handling difficult caregiving tasks (i.e., bathing, helping with toilet activities, and changing clothes). To the best of our knowledge, studies that provide insight into what kind of nursing care participation is associated with working status among family CGs are lacking. After the Long-Term Care Insurance (LTCI) Act was launched by the Ministry of Health, Labor, and Welfare (MHLW) in Japan in 2000, handicapped elderly people aged 65 years or older could access services, which include institutional, home and community-based services [14]. Given the aging of society and the surge in the numbers of elderly people who require care, it is important to adequately and proactively support members of the working-age population who care for their relatives.

This cross-sectional study aimed to investigate the working status of family CGs for people with dementia and to analyze related sociodemographic factors, including care-related stress associated with working status. The study used a nationally representative sample of the Japanese population derived from the nationwide 2013 Comprehensive Survey of Living Conditions (CSLC), which was conducted by the MHLW of Japan.

## Methods

### Ethical consideration

This study was approved by the ethics review committee of the National Center of Neurology and Psychiatry (approval number: A2017-001). Because we did not use any personally identifiable information and based on the Statistics Act in Japan, consent to participate was not required.

### Participants

The present study utilized data from the ‘health questionnaire’, ‘household questionnaire’ and ‘long-term care (LTC) questionnaire’ of the CSLC in 2013, which was a nationwide cross-sectional survey conducted by the MHLW in June-July 2013. The CSLC in 2013 randomly selected 5,530 enumeration districts (EDs) from areas of the 2010 Population Census [15]. The health questionnaire and household questionnaire were distributed to 295,367 households, and members of 234,383 households completed questionnaires. The respondents were all household members, except for individuals who did not live at home during the survey period. The CSLC in 2013 also collected the LTC questionnaire (n = 6,342) from 2,500 randomly selected EDs from the original 5,530, and this questionnaire was distributed to all households with a member who was officially approved for the need for LTC at the time of the survey.

**Fig 1:**
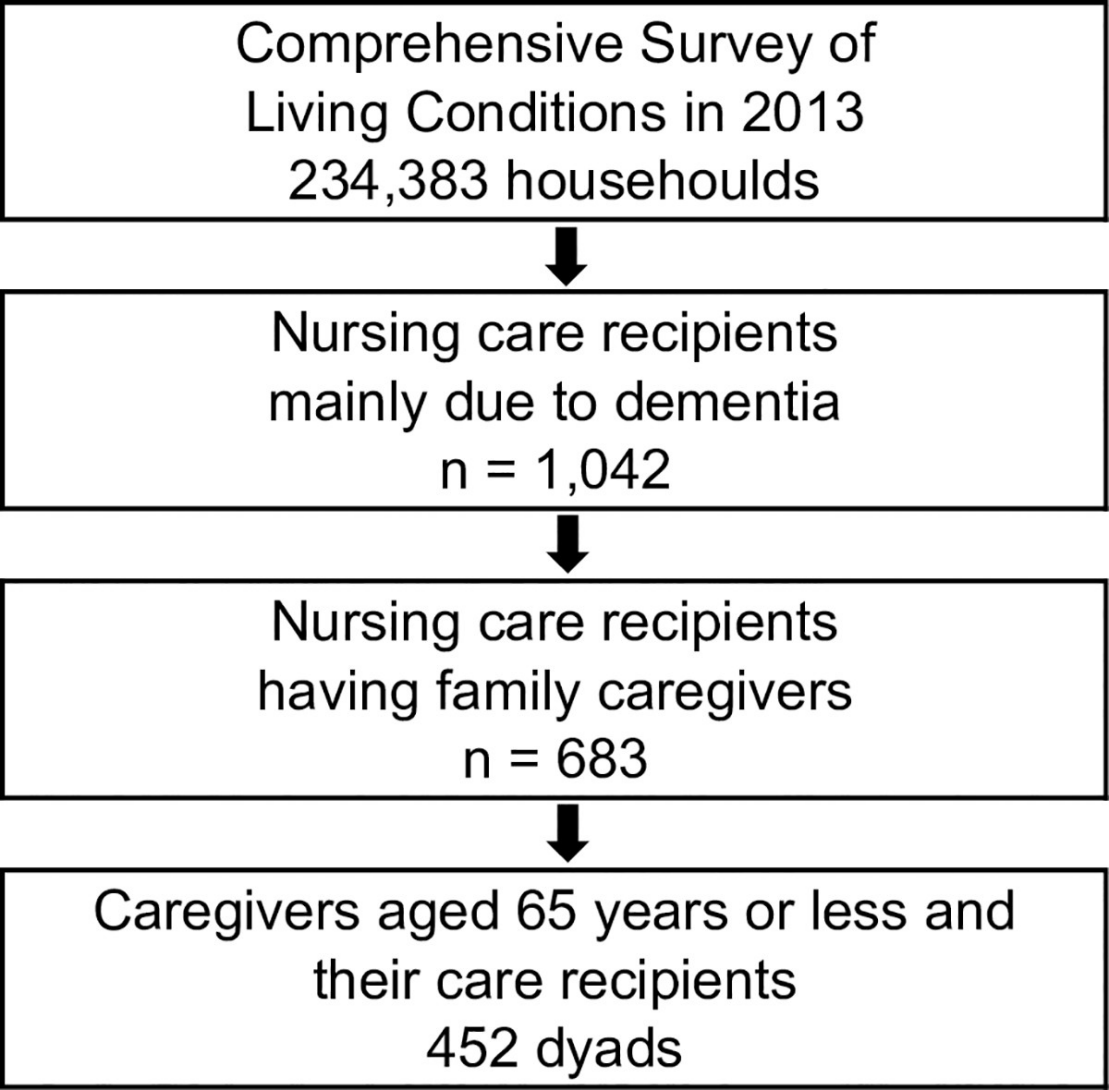
Flow chart of study sample.

The flow chart (Fig 1) indicates how samples were extracted for our quantitative analysis. First, we extracted nursing care recipients whose main reason for receiving care was dementia as care recipients with dementia (CRDs) from the LTC questionnaire of the CSLC (n = 1,042). Under the Japanese LTCI scheme, individuals certified by the municipal government as needing care or support are eligible to receive insurance benefits. Second, we identified the main CGs for the abovementioned household members based on the household questionnaire and included only 683 CRDs with family CGs because this study was focused on the working status of CGs. Third, CGs aged 65 years or less were extracted. This study included 452 dyads of CRDs with dementia and their main CGs as the study population.

We obtained permission to use certain data from the 2013 CSLC for purposes other than those originally intended by the MHLW according to the Statistics Act, Article 33.

### Measures

Regarding nursing CRDs, we obtained data on age, sex, and level of LTC required (7 levels including support required) based on the LTCI scheme.

With respect to the main CGs, we used data on age, sex, relationship with the CRD (spouse, offspring, offspring spouse, other), marital status (married, never married, divorced/widowed), and educational attainment (less than vocational college, vocational college or above). As an assessment of CGs’ working status, having any type of paid work in May 2013 was classified as ‘with paid work’, and not having any type of paid work in the same period was classified as ‘without paid work’. Additionally, other CG characteristics, such as having someone to consult (as a means of social support), knowing how to access consulting services (as health literacy), time spent on nursing care (almost all day, other), participation in nursing care (face washing, brushing teeth, bed bath, shampoo, changing clothes, bathing, changing body position/daily living, helping with toilet, fixing meals (including cooking) and cleaning up, assistance with eating, helping with medication, walking, cleaning up, laundry, shopping, conversation), having subjective symptoms for a few days, psychological distress assessed by the Kessler 6 (K6) scale [16], and visiting hospitals regularly, were extracted from the 2013 CSLC. We used the Japanese version of the K6 scale of psychological distress [17]. In line with the recommended K6 cutoff point, participants with total scores of ≥13 were defined as having a general psychological distress level of serious mental illness, while a score from 0 to 12 suggested no such distress [18].

As household factors, we used data concerning the number of family members at home, house ownership, total household monthly expenditure, and other family members in need of nursing care.

### Statistical analysis

Descriptive analyses were performed to investigate the demographic and clinical variables. To compare the main demographic and clinical characteristics between CGs with and without paid work, an unpaired Student's t-test was performed to analyze the continuous variables, and a chi-square test was performed to analyze the categorical variables. The data are presented as the mean ± SD. Multivariable logistic regression analysis using a forward selection method based on the score test (selection criteria: p<0.05) was conducted with CGs’ working status as the dependent variable and factors related to the CRDs (age, sex, and level of LTC required), factors related to the CGs (age, sex, relationship with CRD, marital status, educational attainment, having someone to consult with, knowing how to access consulting services, time spent on nursing care, participation in nursing care, having subjective symptoms for a few days, psychological distress, and visiting hospitals regularly), and household factors (number of family members at home, house ownership, total household monthly expenditure, and other family members in need of nursing care) as independent variables. Goodness-of-fit for the estimated logistic model was evaluated with the c statistic and Hosmer-Lemeshow test. Missing data were discarded in each analysis, and 9.5% (43/452) of our participants were deleted for the logistic regression analysis. A value of p < 0.05 was considered significant. The data were analyzed using SAS Version 9.4 (SAS Institute Inc., Cary, NC, USA).

## Results

Overall, the mean age of the CGs was 57.1 ± 6.8 years, and the frequency of having paid work among CGs was 57.5% (260/452). The analysis of the influence of sociodemographic data by working status (Table 1) showed that CGs with paid work were significantly younger, spent less time on nursing care, participated less in nursing care (bed bath, shampoo, changing body position/daily living, helping with toilet, fixing meals and cleaning up, assistance with eating, helping with medication, walking, cleaning up, laundry, shopping, and conversation), were less likely to have had subjective symptoms for a few days, and visited hospitals less regularly than those without paid work. In addition, CGs who were male and had higher educational attainment, and their own house were more likely to have paid work.

**Table 1:** Sociodemographic characteristics by working status.

				with paid work		without paid work		p value
Nursing care recipients								
	Age			86.4 ± 4.8	(n = 260)	85.9 ± 6.5	(n = 192)	0.375
	Sex (being male)			16.9%	(44/260)	17.7%	(34/192)	0.827
	Levels of LTC required							
		Support Level 1 or 2, or LTC Level 1		37.4%	(96/257)	36.5%	(70/192)	0.862
		LTC Level 2 or 3		44.0%	(113/257)	46.4%	(89/192)	
		LTC Level 4 or 5		18.7%	(48/257)	17.2%	(33/192)	
Family caregivers								
	Age			55.9 ± 6.8	(n = 260)	58.6 ± 6.3	(n = 192)	<0.001
	Sex (being male)			34.2%	(89/260)	22.9%	(44/192)	0.009
	Relationship with nursing care recipients							
		Spouse		0.8%	(2/260)	3.6%	(7/192)	0.129
		Offspring		51.5%	(134/260)	54.2%	(104/192)	
		Offspring spouse		43.8%	(114/260)	38.0%	(73/192)	
		Others		3.8%	(10/260)	4.2%	(8/192)	
	Marital status							
		Married		81.1%	(196/260)	77.5%	(134/192)	0.381
		Never Married		9.6%	(34/260)	11.8%	(33/192)	
		Divorced/ Widowed		9.3%	(30/260)	10.7%	(25/192)	
	Educational attainment							
		Vocational college or above		26.9%	(70/260)	16.1%	(31/192)	0.007
	Having someone to consult with			96.2%	(250/260)	94.8%	(182/192)	0.486
	Knowing how to access consulting service			99.2%	(258/260)	97.9%	(188/192)	0.473
	Spending almost all day for nursing care			15.0%	(39/260)	32.8%	(63/192)	<0.001
	Participation of nursing care							
		Face washing		13.8%	(36/260)	20.8%	(40/192)	0.050
		Brushing teeth		15.4%	(40/260)	21.4%	(41/192)	0.102
		Bed bath		8.8%	(23/260)	18.2%	(35/192)	0.011
		Shampoo		9.6%	(25/260)	16.1%	(31/192)	0.037
		Changing clothes		29.6%	(77/260)	35.4%	(68/192)	0.192
		Bathing		10.0%	(26/260)	15.1%	(29/192)	0.050
		Changing body positon/ daily living		8.5%	(22/260)	17.2%	(33/192)	0.005
		Helping with toilet		12.3%	(32/260)	23.4%	(45/192)	0.002
		Fixing meals and cleaning up		46.9%	(122/260)	64.6%	(124/192)	<0.001
		Assistance with eating		14.2%	(37/260)	22.9%	(44/192)	0.017
		Helping with medication		48.1%	(125/260)	63.5%	(122/192)	0.001
		Walking		10.8%	(28/260)	24.5%	(47/192)	<0.001
		Cleaning up		47.7%	(124/260)	63.0%	(121/192)	0.001
		Laundry		56.2%	(146/260)	76.0%	(146/192)	<0.001
		Shopping		55.4%	(144/260)	69.8%	(134/192)	0.002
		Conversation		29.2%	(76/260)	45.8%	(88/192)	<0.001
	Having a subjective symptoms in a few days			32.3%	(83/257)	48.1%	(90/187)	0.001
	High psychological distress (K6 ≥ 13)			4.0%	(10/252)	7.9%	(14/178)	0.083
	visiting hospitals regularly			41.0%	(105/256)	60.4%	(113/187)	<0.001
Households								
	Number of family members at home			3.8 ± 1.3	(n = 260)	3.6 ± 1.3	(n = 192)	0.079
	Other family members in need of nursing care			10.0%	(26/260)	14.6%	(28/192)	0.138
	Having own house			96.9%	(252/260)	88.5%	(170/192)	<0.001
	Total household monthly expenditure			33.6 ± 59.5	(n = 250)	27.9 ± 35.1	(n = 182)	0.242

Data are presented as the mean ± SD. LTC; Long-Term Care

To assess the predictors of working status in CGs, we performed a multivariable logistic regression analysis with a forward selection method (Table 2). In that analysis, being male and having a, higher level of educational attainment, and their own house were associated with having paid work. Being older, spending almost all day performing nursing care, participating in helping with toilet activities and laundry, and visiting hospitals regularly were related to not having paid work. The results of the c statistic and Hosmer-Lemeshow test were c = 0.755 and p = 0.349, respectively. These results suggest that the goodness-of-fit for the estimated logistic model was passable.

**Table 2:** Factors associated with the working status of family caregivers for people with dementia.

				Odds ratio (95% Confidence interval)			Wald value	p value
Family caregivers								
	Age			0.94	(0.91	- 0.98)	8.59	0.003
	Sex (being male)			2.52	(1.43	- 4.42)	10.26	0.001
	Marital status							
		Married		Reference				0.031
		Never Married		0.39	(0.19	- 0.80)	6.63	0.010
		Divorced/ Widowed		1.06	(0.52	- 2.16)	0.02	0.876
	Educational attainment							
		Vocational college or above		1.77	(1.00	- 3.11)	3.90	0.048
	Spending almost all day for nursing care			0.32	(0.18	- 0.56)	16.37	<0.001
	Participation of nursing care							
		Helping with toilet		0.53	(0.29	- 0.97)	4.17	0.041
		Laundry		0.52	(0.32	- 0.85)	6.70	0.010
	Visiting hospitals regularly			0.42	(0.27	- 0.66)	14.01	<0.001
Households								
	Having own house			3.65	(1.34	- 9.92)	6.41	0.011

CGs; caregivers, CRDs; care recipients with dementia

Multivariate logistic regression analysis using a forward selection method was carried out with CGs’ working status as the dependent variable and CRDs' factors (age, sex, and level of LTC required), CGs' factors (age, sex, relationship with CRDs, marital status, educational attainment, having someone to consult with, knowing how to access consulting service, time spent for nursing care, participation in nursing care, having subjective symptoms in a few days, psychological distress, and visiting hospitals regularly), and household factors (number of family members at home, house ownership, total household monthly expenditure, and existing other family members in need of nursing care) as independent variables.

## Discussion

This study investigated the potential associations between working status and sociodemographic factors, including nursing care-related factors, among working-age CGs for people with dementia. The majority of working-age CGs were female, educated at less than a vocational college level, and the offspring of CRDs. More than half of CGs aged 65 years or less had paid work. Having paid work was associated with younger, male sex, being married, higher educational attainment level, not spending almost all day on nursing care, not participating in helping with toilet activities and laundry, not visiting hospitals regularly, and house ownership in a multivariable logistic regression analysis.

In this study population, CGs were, on average, in their late 50s, and older age was related to not having paid work. The Japanese Labour Force Survey, which showed that the number of employed persons declines among individuals of higher age or female gender among participants aged 45 years or older [19], corresponds to our results. The majority of working-age CGs were female, and never having been married, was associated with not having paid work. However, the Labour Force Survey, which indicated that never- females who had never been married had a higher proportion in the work force than married females [19], did not support our results. As opposed to the general trend, when elderly family members need nursing care, other members who do not have paid work or who have never married might be expected to take the CG role. Japanese Labour Statistics show that higher educational attainment is associated with lower unemployment [20]. A study concerning working CGs in Japan showed that higher educational attainment (vocational school or above) was associated with lower absenteeism [5]. Work productivity due to higher educational attainment might protect the employment of working CGs. Our results might indicate an unequal distribution of CGs’ burden despite available paid caregiving services under the LTCI scheme. A recent study among middle-aged Japanese women showed that participants’ low education and nonmarried status were associated with a higher likelihood of becoming a primary CG of a severely disabled elderly person even after adjusting for economic status [21].

Our results indicate that spending almost all day on nursing care, which indicates an absence of relief, is associated with not having paid work among CGs. Withdrawal from paid work results in income loss; nevertheless, working-age CGs for the elderly adjusted their working status based on the demands of their caregiving responsibilities, and this adjustment substantially reduced the probability of employment [22, 23]. The ratio of having paid work did not differ by the levels of LTC required based on the Japanese LTCI scheme in our study. However, a study from Germany showed that the severity of dementia assessed by the clinical dementia rating was positively associated with total caregiving time and mainly affected family caregiving time [24]. Differences in assessments of dementia or insurance systems could explain the abovementioned results. In addition, as dementia progresses, the proportion of care provided by paid CGs could increase relative to the time spent by family CGs [25]. Therefore, the usage of formal caregiving could also affect family CGs’ time spent on nursing care.

Although toileting difficulties, including incontinence, might still be considered taboo [26, 27], dementia is an independent risk factor for toileting difficulties even after adjusting for confounders [28]. Our study indicated that CGs’ participation in helping with toilet activities and laundry was negatively associated with having paid work. Participation in these two types of nursing care might make it difficult for CGs to balance work and caregiving for people with dementia. The dependence of elderly CRDs and their behavioral disorders often result in institutionalization. [29]. A frequent CG complaint at the time of institutionalization is incontinence. Caregiving for elderly people in need of help with toilet activities might prevent CGs from engaging in paid work. No previous studies have reported the effect of laundry as nursing care on CGs’ burden or working status. However, laundry related to cleanup after helping with toilet activities might also be a burden on CGs.

Family CGs tend to have not only more mental health problems but also more physical health problems compared to other family members [30, 31]. Such problems could cause CGs to consult healthcare systems or hospitals for their own treatment. Health problems that require regular hospital visits might decrease the physical and mental capacity of CGs, resulting in the CGs' lack of paid work. Physicians should pay attention to CGs’ health problems with regard to the sustainability of their caregiving role.

Although there are differences in house ownership between urban and rural areas, 61.2% of households in Japan own their homes [32]. Previous studies conducted in Japan have shown an association between house ownership and institutionalization [33, 34]. A recent study from Switzerland indicated that house ownership was related to reduced subjective CG burden [35]. In comparison to expenditure, income is an indicator of monthly earnings, and house ownership might be an indicator of household wealth [36]. Furthermore, CGs of family members with dementia might have to prepare to make their own home safer and easier with regard to caregiving.

The current study has several limitations. First, the information regarding CRD characteristics was limited. This study focused on persons with dementia with regard to the severity of care needs. However, the assessment of dementia was based only on the levels of LTC required based on the Japanese LTCI scheme. In particular, our study could not investigate the effect of behavioral and psychological symptoms of dementia (BPSD). The CRDs may have had agitation, anxiety, apathy, disinhibition, delusions, hallucinations, and sleep or appetite changes. Furthermore, we did not obtain information on medical care at home, such as medications, tubal feeding, and suctioning. Future studies should collect a more detailed medical history regarding the underlying BPSD and medical care required at home. Second, this study was limited by its cross-sectional design. Thus, we could not determine a causal relationship between care-related stress factors and working status among the CGs in our study population. A follow-up survey is needed to investigate these associations. Finally, the study sample size was relatively small, although the current study only targeted people with dementia in need of care, and the data were obtained from a nationally representative survey.

In conclusion, several sociodemographic factors, including nursing care-related factors, are associated with the employment status of CGs. Further research should examine detailed information on CRDs’ activities of daily living, BPSD, medical service use, and social support to strengthen the system of support services for both CRDs and their CGs.
